# Retirement intentions of dentists in New South Wales, Australia

**DOI:** 10.1186/1478-4491-8-9

**Published:** 2010-04-01

**Authors:** Deborah Schofield, Susan Fletcher, Sue Page, Emily Callander

**Affiliations:** 1 NHMRC Clinical Trials Centre, Sydney Medical School, University of Sydney, 92-94 Parramatta Rd, Camperdown, NSW, 1450 Australia; 2 NRUDRH, School of Public Health, University of Sydney, 61 Uralba St, Lismore, NSW, Australia

## Abstract

**Background:**

The Australian dental workforce is ageing and current shortages have been predicted to worsen with the retirement of the growing contingent of older dentists. However, these predictions have been based on retirement trends of previous generations and little is known about the retirement intentions of today's older dentists.

**Methods:**

The Dentist Retirement Intentions Survey was mailed to 768 NSW Australian Dental Association members aged over 50 and achieved a response rate of 20%. T-tests, ANOVAs and multivariate regression were used to analyse the data.

**Results:**

On average, participants intend to retire at the age of 66, although they would prefer to do so earlier (p < 0.05). Those intending to leave the workforce within the next 5 years represent 43%. The most common reasons dentists expect to retire are to have more leisure time, to be able to afford to stop working, and job stress or pressure.

**Conclusions:**

The current generation of older dentists intends to retire later than their predecessors. Most wish to remain involved in dentistry in some capacity following retirement, and may assist in overcoming workforce shortages, either by practising part time or training dental students.

## Background

In line with many professions within Australia, the dental workforce has aged significantly since 1986 [1]. With the majority of dentists aged in the 40-48 year age group, this pattern is expected to continue [2], and by 2026 around half of the current practicing dentists will have retired [1]. This ageing of the dental workforce has been flagged as a limitation to meeting the increasing demand for dental care [3].

The ageing of the Australian dental workforce and the predicted worsening of current workforce shortages has been well documented in recent years [1,3-5]. However, these predictions have been based on the retirement patterns of past cohorts of dentists. Workforce demographics have changed substantially over the last two decades [1,3-5]. There has been an increase in the proportion of female dentists, and older dentists are constituting an increasing proportion of the workforce [1,6].

As such, past trends may not tell us the whole story about the future. For example, female dentists generally retire earlier than their male counterparts. Furthermore, the view of many older workers in the past has been that retirement is not the end, but rather the next phase of working life; a chance to explore a new career, enjoy a shift in the work-life balance, and pursue other interests [1,7]. Thus, the work-to-retirement transition intentions of dentists may not be straightforward.

With little known about the work patterns and retirement intentions of today's older dentists, we are currently ill-informed as to how and when the retirement of the baby boomers will affect the capacity of the dental workforce. This paper is the first in a series reporting results of a study that was designed to address this deficit in knowledge.

## Methods

### Data used

The Dentist Retirement Intentions Study is a collaborative venture between the University of Sydney and the NSW branch of the Australian Dental Association (NSWADA). In developing a survey instrument for the study, a number of consultations with practising dentists were conducted and the final survey was approved by the University of Sydney Human Research Ethics Committee in February 2007.

The survey collected demographic and practice characteristics; information on dentists' intended age of retirement and expected reasons for leaving the workforce; as well as financial details such as the value of assets and debts, expected sources of funding for their retirement, and superannuation information.

Contact details of 768 dentists aged 50 years or more were obtained from the NSWADA member database; this figure included 50% of older dentists in Sydney and 100% of those located elsewhere in the state. Surveys were sent to these 768 NSWADA members in October 2007 with a reply-paid envelope included; responses were collected until the end of December. A total of 153 dentists returned a completed survey, a response rate of 20%. This is a better-than-average response to a mail out survey from NSWADA.

There can often be differences between the ages at which a person wishes to retire and when they realistically think they will. There can also be different retirement intentions between age groups. A one-sample t-test was used to test differences between the age at which dentists wished to retire and the age at which they thought they realistically would (their intended retirement age). One-way ANOVAs examined differences in intended retirement age between groups. A forward stepwise multivariate regression was used to try to establish what influences dentists to retire, by identifying predictors of intended retirement age. Any missing values were replaced by the mean for that variable. Variables were entered in 5 steps:

• Step 1: demographic variables - age, sex, marital status, number of children, and self reported health status.

• Step 2: partner variables - education, employment, income, and health.

• Step 3: work variables - type of dentistry, weekly hours, and degree of satisfaction with dentistry as a career.

• Step 4: property ownership - practice building and home.

• Step 5: location variables - town size and geographic area of NSW practice is located in.

All statistical analyses were conducted using SPSS v 15 (SPSS Inc., Chicago, 2006) with significance set at p = 0.05.

## Results

### Characteristics of respondents

Of the 153 respondents to the Dentists Retirement Intentions Study, seven were retired and were excluded from the analyses of retirement intentions. The 146 practising dentists who participated in the study had an average age of 57.5 years but ranged from 50 to 75 years old (similar to the national distribution, where according to the ABS Census the average age of dentists aged 50 years and over was about 58 years). The age distribution of dentists in the study was also similar for the national distribution for dentists over 50 years of age according to the ABS Census. In the ABS Census there was 41.1% of dentists aged between 50 and 54 years, and 37% in this age group in the retirement study; 26.7% aged 55-59 years in the census and 29.7% in the retirement study; 16.6% aged 60-64 in the census and 20.3% in the retirement study; 7.4% aged 65 to 69 in the census and 7.3% in the retirement study; and 8.1% aged over 70 in the census and 5.6% aged over 70 in the retirement study. One 124 (85 percent) were male (similar to the national distribution, where according to the ABS Census, 90% of dentists aged 50 years and over are male). According to the ABS Census only 1.9% of dentists aged 55 years and over were female [8]. There were no female specialist respondents in the Dentist Retirement Intentions Study. This indicates that the sample is a good representation of the national dental workforce aged over 50.

The vast majority were married or in a de facto relationship (86%) and had at least one child (95%). Around three quarters or the participants were Australian born. Dentists felt that both they and their partners were in good to excellent health, with only 6% reporting otherwise. More information on the demographic characteristics of the study participants can be found in Table 1.

**Table 1:** Demographic characteristics of older dentists in NSW, Australia (N = 146)

*Demographic variable*		Average (range) or N (%)
Age		57.5 (50-75)

Sex	Male	125 (85%)
	Female	18 (12%)

Marital status	Never married	0 (0%)
	Married/de facto	126 (86%)
	Separated/divorced	13 (9%)
	Widowed	2 (1%)

Number of children	0	7 (5%)
	1	8 (6%)
	2	53 (36%)
	3	40 (27%)
	4 or more	32 (22%)

Age of children	Youngest	22.5 (2-48)
	Oldest	28.1 (4-50)

Country of birth	Australia	112 (76%)
	Other	29 (20%)

Self reported health	Excellent	41 (28%)
	Very good	65 (44%)
	Good	24 (16%)
	Fair	7 (5%)
	Poor	2 (1%)

Reported health of partner	Excellent	41 (28%)
	Very good	46 (31%)
	Good	33 (22%)
	Fair	8 (5%)
	Poor	1 (1%)

***Work Characteristics***		**Average or %**

Practice Characteristics	Generalist	77%
	Specialist	18%
	Other	5%

Employment	Self employed	84%
	Salaried (public)	4.1%
	Salaried (private)	5.5%

Average hours worked	General practitioners	42 hours per week
	Specialists	35 hours per week
	Combined fields	37 hours per week

Job satisfaction	Very satisfied	48%
	Somewhat satisfied	34%
	Neither satisfied or dissatisfied	10%
	Somewhat dissatisfied	7%
	Very dissatisfied	1%

Location	Capital city	43%
	Major urban centre (>100 000)	20%
	Regional city or large town (25 000-100 000)	13%
	Small town (10 000-24 999)	12%
	Small rural community (<10 000)	12%

### Practice characteristics

Around three quarters of the practising dentists in our study worked in general dentistry, while 18% were specialists and a further 5% worked in an 'other' type of dentistry; text explanations revealed this to be typically a combination of general and specialist dentistry, or of either of those and teaching or research. Over one third of dentists (38%) worked as solo practitioners, while 8% worked in public practice. Study participants were for the most part self-employed (84%), with only a minority employed under salaried arrangements in either the public or private sector (4.1% and 5.5% respectively).

Specialists reported working more hours than either general practitioners or those working in combined fields: an average of 42 hours per week compared to 35 and 37 hours respectively. However, only 76% of specialists' working week was spent on patient care, while general dentists spent 85% of their time with patients.

### Variation in retirement intentions

**Figure 1:**
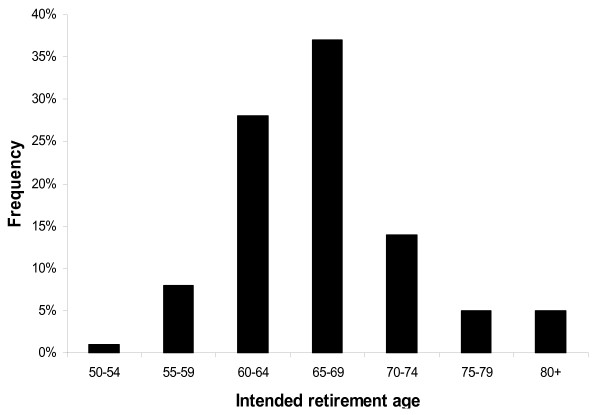
**Intended retirement age of dentists in five year age groups**.

Based on this survey, on average, dentists in NSW would ideally like to retire at age 64. However, they intended to retire at age 66. On average, dentists in New South Wales intend to retire at the age of 66, although would do so at age 64 if they had the choice (p < 0.05). Only 9% of survey respondents thought that they would retire before the age of 60 (Figure 1). Forty-three per cent of dentists aged 50 years and over intended to leave the workforce within five years, while almost three-quarters intend to stop working by 2018.

Dentists with children intend to retire significantly later than those without children (p < 0.05). Dentists with more children intended to retire later than those with fewer children; however this difference was not significant. Male dentists intended to work for slightly longer than their female counterparts (intending to retire at 66 and 63 respectively), and general dentists for longer than specialists (66 and 64 respectively) (Table 2), although these differences were not significant. Several dentists indicated a desire to continue working until the age of 80 or older (5%), with one respondent commenting that as long as he remained in good health, he would never retire.

**Table 2:** Average age of intended retirement in NSW, Australia, by demographic variables

Demographic variable		Average age of retirement (range)
Sex	Male	65.57 (52-90)
	Female	63.33 (51-76)
Marital status	Married/de facto	65.23 (51-90)
	Separated/divorced	65.62 (58-76)
	Widowed	66.00 (65-67)
Children	Yes	61.50 (51-80)
	No	65.78 (56-90)
Country of birth	Australia	65.33 (51-90)
	Other	65.07 (55-80)
Partner employment	Works full time	65.07 (51-85)
	Works part time	65.23 (55-90)
	Not in paid employment	65.78 (58-80)
Type of dentistry	General	65.55 (51-90)
	Specialist	61.04 (60-73)
	Other	65.43 (55-70)
Town size	Capital city	65.40 (56-80)
	>100 000	65.80 (55-80)
	25 999-100 000	64.21 (57-76)
	10 000-24 999	64.33 (52-77)
	<10 000	65.27 (51-90)

Study participants were asked to indicate which of a number of possibilities they predicted would be the reason for their future retirement (Table 3). For all dentists, the most common responses were to have more leisure time (51%), being able to afford to stop working (49%) and job stress or pressure (23%) (multiple responses could be selected and there was no ranking of the responses).

**Table 3:** Predicted reasons for retirement among dentists and average intended age of retirement (in NSW, Australia)

	**% of dentists of each sex/work type (expected age of retirement)**^**a**^					
	**General dentists**		**Specialists**	**All dentists**		

**Reason for retirement**	**Male** **N = 95**	**Female** **N = 14**	**Total** **N = 25**	**Male** **N = 124**	**Female** **N = 18**	**Total** **N = 146**

I can afford to	53 (65.0)	50 (58.9)	52 (63.9)	51 (64.8)	50 (59.1)	49 (64.0)

Superannuation rules making retirement financially advantageous	19 (65.0)	21 (62.3)	12 (61.7)	17 (64.5)	22 (63.0)	17 (64.3)

Becoming eligible for the old age pension	1 (69.0)	7 (76.0)	0	1 (69.0)	6 (76.0)	1 (72.5)

Spouse retiring	1 (58.0)	7 (58.0)	0	1 (58.0)	11 (58.0)	2 (58.0)

Spouse wanting me to retire	8 (63.1)	7 (75.0)	8 (62.5)	8 (63.0)	6 (75.0)	8 (64.1)

Spouse's income sufficient	0	0	0	0	0	0

To spend more time with family	17 (64.4)	29 (69.0)	12 (65.0)	16 (64.7)	22 (69.0)	16 (64.4)

To have more leisure time	52 (64.9)	57 (65.4)	52 (63.1)	52 (64.6)	61 (64.8)	51 (64.7)

Lack of interesting work/boredom	6 (63.2)	0	0	5 (63.2)	0	4 (63.2)

Job stress/pressure	25 (63.5)	36 (62.4)	4 (60.0)	22 (63.8)	39 (61.7)	23 (63.4)

Accepting voluntary redundancy	1 (68.0)	0	0	1 (68.0)	0	1 (68.0)

Own ill health	8 (70.0)	29 (68.0)	4 (65.0)	8 (69.4)	28 (67.4)	10 (68.6)

Ill health of family member	0	14 (70.0)	0	0	11 (70.0)	1 (70.0)

^a^Note: there were three work types recorded in the survey (general dentists, specialists and other dentists). Only the disaggregated results for general dentists and specialists are presented here, along with those for all dentists. All specialists responding to the survey were male.

Males were more likely than females to expect to retire due to a lack of interesting work, with no female dentists indicating that this would be a reason for retirement. Females more often predicted their retirement to be associated with ill health (Table 3). Female dentists expecting to retire because their spouse wanted them to predicted a significantly older retirement age than males retiring for the same reason (p < 0.05), while the reverse is true for dentists who envisage themselves leaving the workforce because they can afford to do so (p < 0.05). Other potential reasons for retirement suggested by the study participants include bureaucracy, moving with the family to further children's' education, to pursue other interests, a desire to stop working before skills start to fade, and a belief that the physical demands of the job are such that to work beyond a reasonable age puts patients at risk.

A forward stepwise multivariate regression analysis revealed that of a number of demographic, work, and location variables entered, only age and home ownership were significant predictors of intended retirement age. Together, these two variables accounted for just under one third of the variance in intended retirement age, with 27% of variance attributable to current age (p < 0.05) and home ownership responsible for a further 2% (p < 0.05). This suggests that paying off debt is an important determinant of retirement.

### Semi-retirement

**Figure 2:**
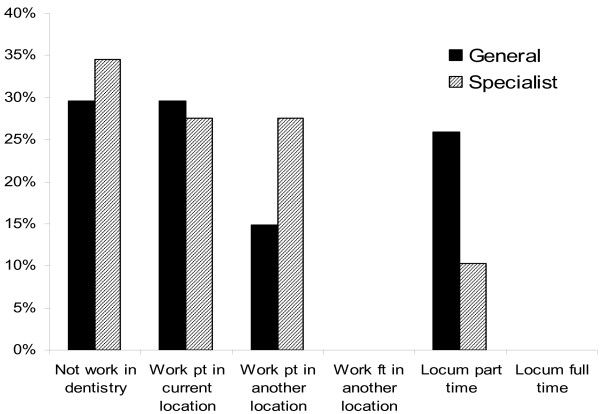
**Post-retirement plans of general and specialist dentists**.

Some dentists transition into retirement via part-time work. Two-thirds of the dentists in this study worked in a practice where part time work was available. Of these, 71% were either currently working part time or considering part time work in the future. Of the dentists for whom part time hours were not available in their current practice, two thirds reported that they would like the option. Part time work appears to be an attractive alternative to full retirement, with 54% of survey respondents declaring a desire to continue working at reduced hours at either their current location or in another practice after ceasing full time work. Thirty-seven per cent of dentists intend not to work in dentistry at all following retirement from their current practice, while 27% plan to work as a locum part time. A comparison of the post-retirement plans of general and specialist dentists can be found in Figure 2.

### Policy impacts

The changes to superannuation arrangements in 2006 appear to have had little effect on the retirement plans of older dentists in NSW, with 57% reporting that there would be no change to their intended retirement date. Of those who predicted the superannuation changes would have some impact, two-thirds thought that they would retire later than previously planned (19% of the total sample). A further 13% were unsure how the new policy would affect their retirement.

## Discussion

The results of this study suggest that while the traditional retirement age of 65 is attractive to many dentists, the traditional concept of retirement is not. The next 10 years will see approximately three-quarters of currently practising older dentists leave the workforce, although the impact of this outflow on current workforce shortages will be mitigated somewhat by the large proportion of retirees intending to continue working in dentistry on a part time basis.

Previous research has found that there is a sense among baby boomers (the generation born between 1946 and 1964) that retirement signifies old age and represents an image of frailty, disempowerment, decay and inactivity [7]. Therefore, delaying retirement is a way of fending off old age and retaining a sense of purpose and status within the community, particularly for higher income earners. Our findings indicate that dentists intend to delay retirement for longer than dentists have in the past, with just 9% of respondents to our survey intending to retire before the age of 60, compared to the 16% of older dentists who did so twenty years ago [1]. This may partly be due to changes to superannuation policy, which effectively provides for a tax-free income stream for those who work to the age of 60 years [9]. Similarly, dentists were found to generally work fewer hours per week than other health care professionals [10,11], which may also explain why dentists are prepared to delay full or partial retirement.

Another possible explanation for the prolonging of retirement is the changing in dental practices that has occurred over the last decade. There is now a greater emphasis on preventative practices and less on extraction and replacement [12,13]. The investment in new technologies associated with this change in practice may require dentists to extend their expected retirement date to obtain value from their investment.

### Changing concepts of retirement

That retirement is a time for changes in work and recreation practices would certainly seem to be the case for the participants in the current study, for whom having more leisure time was the most commonly cited intended reason for retirement (see Table 3). In contrast, a study of public sector employees in NSW found that financial security was the most important influence on their intended retirement date, with only 30% indicating that pursuing leisure activities was a 'very important' factor in determining when to retire [14]. This difference may reflect a difference in income between the two samples, as financial issues have been found to be less of a consideration for higher income earners when contemplating retirement [7].

With many older workers rejecting the traditional notion of full retirement, part time work (or semi-retirement) is an attractive option for those who wish to have more time for other activities but who do not want to leave the workforce altogether [7,14-16]. Of the dentists in this study, only thirty-seven per cent indicated that retirement from their current position would mean complete retirement from dentistry; the remainder expressed an interest in semi-retirement and continuing to work on a part time or locum basis, as shown in Figure 2. Almost one quarter of our sample did not have the option of part time hours in their current location but wished they did. Options to increase the availability of part time work should therefore be considered in order to prevent these dentists from being essentially forced into full retirement, therefore exacerbating the current widespread shortages in dentistry [6]. For example, a register of dentists available to work part time might be one way of covering leave

Semi retirement and practising at reduced hours is not the only alternative to retirement for older dentists. Teaching, research, and administrative careers are other possible ways to contribute to the field of dentistry while retiring as a practitioner, and may be particularly attractive to older dentists who feel that the effects of ageing are such that continuing to practise would potentially place patients at risk, but who do not wish to give up their career entirely [17]. This situation may become more and more common as increasing numbers of dentists push their retirement further into old age. Encouraging potential retirees to consider teaching as a new career direction is also likely to enhance the sustainability of dentistry into the future; with more teachers, dental school numbers can be increased, thus improving the future capacity of the dental workforce to meet demand.

### Increasing student numbers: not the short-term solution

The effect of increased student numbers will not filter through to the workforce for at least half a decade, however. With almost half of currently-practising older dentists aged 50 years and over intending to cease full time work within the next five years, retention of some of these individuals is essential in order to maintain workforce capacity in the short term. Past research has found that the vast majority of people can be persuaded to retire later if appropriate inducements are offered [15]. The importance of improving the availability of part time work has already been discussed; surveys of employees in a number of sectors have revealed that many could be persuaded to work past their intended retirement date if they could reduce their hours without affecting superannuation entitlements [14,15]. The large proportion of dentists considering locum work during their semi-retirement is encouraging, however increasing locum numbers would seem to be an expensive option for improving overall workforce capacity [15]. Conducting interviews to examine retirement preferences and whether and how an individual might be tempted to remain in the workforce has been suggested to overcome shortages created by the retirement of older psychiatrists [15], and it is recommended a similar initiative be considered to enhance retention of our older dentists.

## Conclusions

In conclusion, the intended retirement age of dentists appears to be later than previous data suggests. The traditional notion of retirement does not appear to appeal to many older dentists, and although they look forward to having more leisure time and spending more time with family, most also wish to remain involved in dentistry in some capacity. This is good news when considered in the context of previous predictions of worsening shortages in the profession; however the continued workforce participation of older dentists should not be taken for granted. Providing a forum for these individuals to discuss their options as they move towards retirement may enable specific incentives to be offered in order to encourage a later retirement date.

## Competing interests

The authors declare that they have no competing interests.

## Authors' contributions

DS designed and led the study; SF prepared the surveys and carried out the analysis; and SP was involved in the conception of the study and, along with EC, contributed to the manuscript. All authors read and approved the final manuscript.
